# Improving the experience of dementia and enhancing active life - living well with dementia: study protocol for the IDEAL study

**DOI:** 10.1186/s12955-014-0164-6

**Published:** 2014-11-30

**Authors:** Linda Clare, Sharon M Nelis, Catherine Quinn, Anthony Martyr, Catherine Henderson, John V Hindle, Ian R Jones, Roy W Jones, Martin Knapp, Michael D Kopelman, Robin G Morris, James A Pickett, Jennifer M Rusted, Nada M Savitch, Jeanette M Thom, Christina R Victor

**Affiliations:** Research in Ageing and Cognitive Health, School of Psychology, Bangor University, Bangor, UK; Personal Social Services Research Unit, London School of Economics and Political Science, London, UK; School of Medical and Health Care Sciences, Bangor University, and Betsi Cadwaladr University Health Board, Bangor, UK; Wales Institute of Social and Economic Research, Data and Methods, Cardiff University, Cardiff, UK; Research Institute for the Care of Older People, Bath, UK; Department of Social Policy, London School of Economics and Political Science, London, UK; Department of Psychological Medicine, King’s College London Institute of Psychiatry, London, UK; Department of Psychology, King’s College London Institute of Psychiatry, London, UK; Alzheimer’s Society, London, UK; School of Psychology, University of Sussex, Sussex, UK; Innovations in Dementia, Dementia, UK; School of Medical Sciences, University of New South Wales, Sydney, Australia; Department of Clinical Sciences, Brunel University, London, UK

**Keywords:** Quality of life, Life satisfaction, Well-being, Person with dementia, Carer, Alzheimer’s disease, Vascular dementia, Fronto-temporal dementia, Parkinson’s disease dementia, Lewy body dementia

## Abstract

**Background:**

Enabling people with dementia and carers to ‘live well’ with the condition is a key United Kingdom policy objective. The aim of this project is to identify what helps people to live well or makes it difficult to live well in the context of having dementia or caring for a person with dementia, and to understand what ‘living well’ means from the perspective of people with dementia and carers.

**Methods/Design:**

Over a two-year period, 1500 people with early-stage dementia throughout Great Britain will be recruited to the study, together with a carer wherever possible. All the participants will be visited at home initially and again 12 months and 24 months later. This will provide information about the way in which well-being, life satisfaction and quality of life are affected by social capitals, assets and resources, the challenges posed by dementia, and the ways in which people adjust to and cope with these challenges. A smaller group will be interviewed in more depth.

**Discussion:**

The findings will lead to recommendations about what can be done by individuals, communities, health and social care practitioners, care providers and policy-makers to improve the likelihood of living well with dementia.

## Background

Measuring and improving general well-being across the population, rather than focusing exclusively on measures of economic performance, is central to the UK Government’s development of social and health policy. Within this broad policy trajectory, enabling people with dementia and their primary (usually family) carers to live well with dementia is seen as a priority [1]. The UK National Dementia Strategy [1] focuses on improving public and professional attitudes and understanding, providing early diagnosis and intervention, and ensuring high-quality care and support through all stages of dementia [2]. Gains in public understanding and effective service provision require a clear articulation of what it really means to ‘live well’ in the context of the challenges dementia brings for individuals, relationships and communities, and a clear and current understanding of the factors that influence the ability to live well with dementia [3].

In the limited instances where the concept of ‘living well’ has been discussed explicitly in the literature relating to dementia, it is equated with experiencing a good quality of life (QoL) [4]. Perceived QoL and quality of care can be regarded as providing important indices of whether a person with dementia or carer is living well with the condition, but they do not capture all the elements involved. Living well with chronic illness and disability has been defined as ‘the best achievable state of health that encompasses all dimensions of physical, mental and social well-being’ [5]. This definition emphasises the pivotal role of social factors in determining the ability to live well, noting that ‘living well is shaped by the physical, social and cultural surroundings, and by the effects of chronic illness not only on the affected individual but also on family members, friends and carers’. It also acknowledges the centrality of subjective perceptions and appraisals: ‘for each individual with chronic illness, to live well takes on a unique and equally important personal meaning, which is defined by a self-perceived level of comfort, function and contentment with life’. Older people in general similarly hold multi-faceted and individual views about what it means to live well in later life [6]. Living well in this definition is a broader construct than QoL, incorporating concepts of well-being and life satisfaction, and reflecting the importance of social capital, assets and resources and the potential for social participation.

Enabling people to live well with chronic illness or disability may reduce the costs to society as well as benefitting individuals, families and communities. In the case of progressive neurodegenerative conditions, enabling people to live well presents particular challenges as needs change over time. In relation to dementia, it has been noted that a shift in perspective from a primarily medical or disease-oriented focus to a more socially-oriented understanding [7] is needed to take account of aspects that have hitherto been largely neglected in research with people with dementia, such as differences in social capital, social resources and social circumstances [8]. The Improving the experience of Dementia and Enhancing Active Life (IDEAL) project will examine how social and psychological factors influence the possibility of living well with dementia, will identify what changes could be made at individual and community levels, and will result in recommendations for social and health care purchasers, providers and planners, and advice and guidance for people with dementia and those who support or advocate for them.

### Theoretical framework

**Figure 1:**
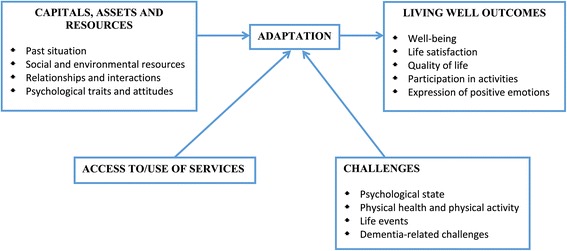
**Overview of factors thought to affect the ability to live well with dementia that will be examined in IDEAL.**

Our investigation will be conducted in relation to a model that identifies the following key elements: *capitals, assets and resources, challenges, adaptation,* and ability to *live well*. This model acknowledges the centrality of subjective evaluations in determining whether a person is ‘living well’ [9]. *Capitals, assets and resources* crucially shape the pathway through the experience of dementia, whether directly affecting symptoms and progression for the people with dementia, or affecting the potential for adaptation for the people with dementia and carers, or both. They can influence the effects of pathology, mitigate the extent of any resulting disability, prevent excess disability and reduce the risk of social exclusion [10]. They include social (e.g. social networks, social contacts, interpersonal relationships, availability of help and support), environmental (e.g. neighbourhood), financial (e.g. income), physical (e.g. physical function and fitness), and psychological (e.g. self-esteem, optimism) aspects, as well as access to social and health care. The operation of these capitals, assets and resources can be affected by the presence and severity of dementia-related and other *challenges* and by the degree of *adaptation* achieved. *Adaptation* is the potential to be able to adapt and to manage and cope with the *challenges* dementia brings, including the symptoms themselves and their impact and implications, as well as any other challenges encountered (e.g. dependence, other health problems, frailty, sensory impairments, depression, carer stress). It is central to the possibility of living well and maintaining QoL [11-13], as indicated by models of ‘successful’ ageing in the face of physical illness, frailty or disability [11]. Adaptation encompasses both practical and social changes, such as modifying activities, modifying the environment, or mobilising additional support [11], and psychological changes, such as altering expectations and revising goals [12]. The outcomes of the complex interactions between *capitals, assets and resources*, *challenges* and *adaptation* are reflected in the social participation, expression of positive emotions, and subjective evaluations of well-being, life satisfaction, and QoL, that together index the extent to which the person is *living well* with dementia. This model is summarised, in simplified form, in Figure 1.

### What do we know about indicators of living well with dementia?

The experience of living well is indexed by positive evaluations of subjective well-being, life satisfaction and QoL. These indices are inter-related but encompass distinct elements. Subjective well-being reflects the emotional response to a situation, including the experience of an appropriate balance of positive and negative emotions [14]. Life satisfaction comprises positive elements of happiness, well-being, a sense of meaning and purpose in life, ability for continued personal growth, a sense of being in control of one’s life, and active social participation [8]. Quality of life is defined by the World Health Organisation as ‘an individual’s perceptions of their position in life in the context of the culture and values systems in which they live and in relation to their goals, expectations, standards and concerns. It is a broad ranging concept, affected in a complex way by a person’s physical health, psychological state, personal beliefs, social relationships and their relationship to their environment’ [15]. Quality of life has been conceptualised either as a generic construct or in terms of aspects related to a specific domain, such as health (Health-Related QoL, HRQoL). While HRQoL should focus specifically on factors affected by the condition [16], given the pervasive effects of dementia, there is in practice often considerable overlap with more generic conceptualisations. For each of these key indices of living well, there are consistent and well-established relationships with the capitals, assets and resources identified in our theoretical model for older people in general. Here we propose to interrogate, for the first time, the relationships between these key determinants of living well from the perspective of people with dementia. We will integrate data on physical and psychological health, disposition, personal beliefs, social relationships and environmental factors to provide a comprehensive model of the relative impact of each element and the trajectory of change as the disease progresses.

Subjective well-being is important for health, and influences health trajectories, although this is more clearly established for physical than for mental health [14]. A recent review emphasises the degree to which socio-economic status, income inequality and cultural differences, along with personality and emotional style, are related to individual variability in well-being, and both giving and receiving social support to others have powerful effects on well-being and health [17]. Social engagement and social support are key aspects of well-being in later life [18] and social participation is associated with better subjective health [19]. Perceived social support and participation in activities are significantly associated with well-being in cognitively-impaired residents [20], while loneliness has a negative impact on well-being in older people [21]. Feelings of loneliness are predictive of developing dementia [22,23]. Loneliness is a significant issue for people with dementia because developing dementia often results in a loss of relationships and social contacts and a reduction in social networks and social engagement [24]. Life satisfaction is lower in those with depressed mood, but higher in those with good social networks and social support, economic security, a strong sense of personal control, and good subjective health [25]. Socio-economic status [26], personality traits [27] and educational level [28] are important correlates or predictors of life satisfaction in older people without dementia. One study of life satisfaction in people with mild to moderate dementia found that social support plays an important role [29]. Recognising the importance of social networks and relationships, there is a need to examine the nature of social contact, engagement and participation for people with dementia, and the implications for well-being across the illness trajectory, to help identify strategies that will encourage communities to include and better support people with dementia.

It has been argued that little is known about the QoL or HRQoL of people living with dementia [30-32]. Quality of life in dementia has been studied in relation to a relatively limited set of factors [8], and numerous studies demonstrate that a large proportion of the variance in QoL scores, whether self-rated by people with dementia or proxy-rated by carers, remains unexplained by commonly-measured patient and carer factors including symptoms, co-morbidity, carer burden, and basic demographic variables [33,34]. Depressed mood is a common predictor of poor QoL scores, but cognitive function, behavioural symptoms and functional ability show no clear associations with QoL [30,33,35]. Even the inclusion of additional factors such as difficulties in the caregiving relationship only accounted for 38% of the variance in QoL scores [36], while including the psychological variables of self-concept, self-reported depression and quality of relationship accounted for 52% of the variance in self-rated QoL-AD scores in the Memory Impairment and Dementia Awareness Study (MIDAS) [35]. There is a need to take a broader perspective if we are to understand variations in QoL among people with dementia [37]. Individual appraisals of QoL are influenced by social processes (e.g. social comparison) alongside psychological factors (e.g. optimism) [38], and adaptation of expectations in response to changing circumstances and experiences may allow for QoL to be maintained despite objective deterioration in circumstances or decline in aspects of functioning [13].

The relevance of social, environmental and cultural factors is strongly emphasised in qualitative accounts outlining the domains which people with dementia themselves consider important in relation to QoL, but these factors have rarely been included within large-scale quantitative studies. The domains described by people with dementia have been characterised as primarily social in nature [39] and include social interaction, social connectedness, social relationships, and meaningful social activities alongside financial security, psychological well-being, autonomy, and health [24,38,40,41]. A recent review of domains relevant to QoL in dementia highlighted the importance of social and psychological factors, including autonomy, choice, control and dignity, alongside environmental, economic and cultural elements [42]. Spiritual well-being has also been cited as a key QoL domain [43]. Social inequalities and the resulting health disparities are likely to impact negatively on QoL [7], while people with more developed social networks report better QoL [44]. There is considerable evidence that strong social networks and social engagement contribute to maintaining cognitive health (mental fitness) in later life [45], and these factors may also have a protective effect for people with established cognitive impairment or dementia [46]. Many studies have measured the quantity of social networks and their association with cognitive health, but it is the quality of networks that is likely to be important, as well as their interrelationship with levels of social capital and social resources [47]. Few studies are able to provide detailed longitudinal data in this area [48]. Social networks may be related to levels of formal and informal (unpaid) support and care. Unmet needs are important predictors of self-rated QoL in people with dementia and social networks appear to be related to unmet needs, which affect QoL [44]. Studies that follow the individual pathways of people with dementia indicate important life events and transitions where the identification of specialist support may be beneficial [49].

While the social environments of people with dementia and the social interactions occurring within those environments are important influences on QoL [50], there is very little research addressing the lived experience of dementia in the context of the physical and social environment or neighbourhood. This is despite evidence that living in a neighbourhood with high levels of deprivation is associated with lower cognitive function, even when controlling for individual socioeconomic circumstances [51], while neighbourhood resources have the potential to promote cognitive reserve [52]. A recent qualitative study, noting the importance of access to a local, familiar, safe environment for well-being, deftly characterised the ‘shrinking world’ of people with dementia in which the area of physical space which people with dementia find it comfortable to occupy becomes gradually smaller [53]. The lack of neighbourhood accessibility and the absence of a feeling of safety are indicative of low social capital [54], and confinement within the home has been identified as an objective indicator of poor QoL along with the absence of any expression of positive emotions [16]. Environmental characteristics and accessibility have major implications for the possibility of continued social participation and hence for the ability to live well with dementia. It is timely to examine these hitherto neglected factors in dementia research. Focusing on the lived experience of dementia and the daily lives, social relations and physical and social environments of people with dementia and carers would make it possible to identify how mundane social interactions in a range of areas (shopping, travelling, leisure, internet, volunteering, financial etc.) facilitate or present barriers to living well.

In addition to the limitations of research focusing on the dimensions of living well with dementia, the trajectory of living well over the course of dementia and the factors associated with change in the ability to live well remain to be clearly established. Using the example of QoL, the most extensively-studied element of our concept, evidence on changes in level of QoL in community-dwelling samples with mild to moderate dementia is inconclusive [55-59] as are findings for care home residents [31,32,60], and all studies note considerable individual variation, described in one case as ‘vast’ [58]. Changes in clinical variables are not directly associated with increases or reductions in QoL score [55,56]. Recent findings support the view that social and interpersonal factors may play a stronger role; for example, a positive relationship with carers plays an important role in maintaining QoL in people with early-stage dementia [59] and a reciprocal relationship between carer factors, such as carer stress and perceived quality of relationship, and observer-rated people with dementia well-being six months later has been reported [61,62]. Well-being in the carer may be a crucial influence on QoL for the person with dementia and such reciprocal influences require consideration. Whilst research has explored the influence of characteristics of the person with dementia, such as behavioural problems, on carer well-being [63], relatively little attention has been paid to how carer factors, such as burden or feelings of competency, impact on the well-being of the person with dementia [61], yet research exploring predictors of placement in full-time care indicate that carer factors play an important role in this decision [64]. It is widely understood that outcomes for carers of people with dementia are very variable [65], although a relatively limited range of carer factors has been studied in relation to outcomes. Examining a wider range of social and psychological factors in a large sample of carers will clarify how capitals, assets and resources influence their ability to address dementia-related challenges and adapt to the carers role, and contribute to the possibility of living well for the people with dementia and for themselves. There is a need to investigate the factors underlying changes in QoL and individual variations in QoL trajectories for people with dementia [58,59] to capture the complex interplay of social and psychological variables involved. This conclusion applies equally to the other components of our concept. Our proposed large-scale longitudinal study of the factors and predictors of living well with dementia and how this changes over time fulfils this need.

### Why is this study timely?

Policy recommendations [3,66] recognise the importance of understanding the personal and social experience of people with dementia and carers. Acknowledgement of the personhood of people with dementia [67] and the importance of interpersonal relationships [68] has brought improvements in care provision, but limited consideration has been given to the way in which wider social issues affect the ability to live well with dementia [69] or to the capitals, assets and resources reflecting the accumulation of advantages or disadvantages over time [70]. Social participation and social networks are central to the accumulation of social capital, and greater social capital is linked with better access to health care and better subjective health at both individual and community levels [71]. Similarly, little emphasis has been placed on the personal psychological characteristics, shaped by social and cultural norms, which contribute to the ability to adapt to and live well with dementia or to the interaction between the condition and age-typical changes and transitions. There is a need to examine not just the subjective experience of dementia, but also its social and environmental context. Finally, although it is understood that people with dementia [72,73] and carers [74] respond in very diverse and individual ways, little is known about how people with dementia and carers make sense of and adapt to the condition and to the changes they experience over time, or about the reciprocal influences between each member of the dyad. The dementia trajectory encompasses enormous variation from the earliest stages to the end of life, and what constitutes living well differs across this trajectory. We will focus on people with dementia who, on entry to the study, are living in their own homes with mild or moderate dementia, and follow them over time, observing whether and how their situation changes, and monitoring the progression of dementia, in order to identify the factors that influence their ability to live well as their dementia progresses. Novel elements in this study are the detailed and extensive focus on forms of capital, assets and resources, the emphasis on the process of adjustment, and the inclusion of multiple indices of ‘living well’.

There are currently no large datasets from cohorts of people with dementia addressing living well with dementia, or explaining how social and psychological factors influence the ability to live well, that can inform policy and practice. Internationally, longitudinal studies of ageing such as the Odense study in Denmark [75] and Cache County [76] studies in the USA, have focused on estimating the prevalence and incidence of dementia and identifying primarily medical and health-related risk factors. The Berlin Ageing Study [77] uniquely adopts a broad interdisciplinary gerontological perspective in its focus on the old and very old, but does not have a primary focus on dementia. In the UK, the Cognitive Function and Ageing Studies (CFAS) study has focused on estimating incidence and prevalence of dementia [78] and identifying risk factors, although it does provide some useful longitudinal socio-economic data on people with dementia. CFAS-2 is currently repeating this exercise in England and Wales, and while CFAS-Wales includes some consideration of the role of social and psychological factors in predicting development of dementia, this does not directly address the nature of what it means to live well with dementia. A small number of studies have investigated the longitudinal trajectory of QoL in dementia, but sample sizes have been relatively small and some have focused only on care home residents [31,32,56-60]. The Dependence in AD in England (DADE) study recently provided data on costs and carer burden with regard to living with dementia [79], again with a relatively small sample. However, there remains a dearth of data addressing the complex inter-relationship between forms of capitals, assets and resources at individual and community levels and the ability to adapt to and live well with dementia. There are no large cohort studies specifically focused on people with dementia that can provide definitive information about these factors.

It is now understood that people with dementia at all stages through to moderately severe impairment can respond to self-report questionnaires [29,80,81], and many people with severe dementia can describe their feelings and experiences and comment on their situation and QoL [38,59,82]. While some studies have suggested that lack of awareness in people with dementia impacts on the reliability of self-ratings of variables such as QoL [83,84], others have not found such an association [59,85,86]. Quality of life is an individual, subjective evaluation of one’s own situation, and self-reports should therefore be prioritised wherever possible. The status of proxy ratings made by carers is questionable [87,88], as people with dementia and carers place different degrees of emphasis on various factors. For people with severe dementia, carer ratings may be an important source of information. One solution is to obtain both types of rating at earlier stages, making it possible to interpret informant ratings more effectively once these become the only ratings available [87]. Another is to identify objective indicators of poor well-being in severe dementia, for example, confinement to home and expression of a restricted range of positive emotions [16,89]. Using a combination of quantitative methods in this way, it is feasible to examine the experience of ‘living well’ across the trajectory of dementia. There is also a need for detailed qualitative studies of the lived experience of dementia, focusing on the impact of the environmental context on the social, physical and psychological aspects of the lives of people with dementia and carers [53,90]. Our study will construct a novel and detailed longitudinal data set that focuses specifically on measures of individual social and cultural capital, socio-economic status, assets and resources, adaptation, community and neighbourhood, life satisfaction and QoL, incorporating a qualitative perspective.

### Study aims

IDEAL is a longitudinal cohort study using a mixed-methods approach to characterise the social and psychological factors that support or constrain the ability of people with dementia and carers to live well with any type of dementia. We will examine the impact of capitals, assets and resources on the ability to live well with dementia. This will result in an action plan intended to assist policy-makers, purchasers and providers to develop evidence-based policies and practices aimed at preventing or reducing unnecessary disability, preserving independence, reducing the economic burden for families and for society, and maintaining well-being across the dementia disease trajectory, in order to allow more people with dementia to live well, and all people with dementia to live better.

### Research questions

Given the conceptual, empirical and methodological limitations of the existing evidence, our central, overarching research questions are as follows:

1. How do *capitals, assets and resources*, and adaptation in response to dementia-related and other challenges, influence the ability to *live well* for people with dementia and carers, and what are the reciprocal influences between people with dementia and carers factors?
2. How do changes over time in *capitals, assets and resources*, dementia-related and other *challenges*, and *adaptation* affect evaluations of *living well* for people with dementia and carers?
3. What do people with dementia and carers believe helps or hinders the possibility of *living well,* and what factors are particularly important to them as regards being able to live well with dementia?

## Method

### Design

This is a mixed-method, longitudinal cohort study of people with dementia and carers. The quantitative arm will answer research questions 1 and 2, and the qualitative arm research question 3. Quantitative assessments will be conducted at three time points: Time 1 (T1), one-year follow-up (Time 2, T2), and two-year follow-up (Time 3, T3). Qualitative assessments will be conducted at T2 and T3 with a sample of participants showing evidence of change in indicators of living well, and will enrich the quantitative findings by illuminating the reasons and subjective experiences underlying these changes. The IDEAL study has been approved by the North Wales Research Ethics Committee - West (reference 13/WA/0405), the Scotland A Research Ethics Committee (reference 14/SS/0010) and the Ethics Committee of the School of Psychology, Bangor University (reference 2014 – 11684). The IDEAL study is registered with UKCRN, registration number 16593.

### Participants

We will recruit 1500 people with dementia over a 24-month period and assess them initially (T1) and on two further occasions 12 (T2) and 24 (T3) months later. We will focus on people with dementia who, on entry to the study, are living in their own homes. There will be no restrictions on age. Sample size has been determined on the basis of our prior experience with and findings from the MIDAS [55,62] and DADE [79] studies and on the nature of our proposed primary statistical analyses using structural equation modelling (SEM) [91]. Inclusion criteria for people with dementia will be a clinical diagnosis of dementia (any sub-type) and a Mini-Mental State Examination (MMSE) [92] score of 15 or above. Recruitment will therefore target people who have mild to moderate dementia on entry to the study, yielding a sample ranging from mild to severe dementia at follow up. To ensure that the study successfully recruits participants across the whole range of mild to moderate dementia we will perform a check on the distribution of T1 MMSE scores six months into recruitment and will stratify later recruitment if necessary. Carers will be the designated primary carers of people with dementia who meet inclusion criteria. For the purposes of the study we consider a carer to be someone who looks after a relative or friend and provides practical or emotional unpaid support. Exclusion criteria for people with dementia will be co-morbid terminal illness at T1, inability to provide informed consent at T1, and any known potential for home visits to pose a significant risk to research network staff. We will seek to recruit a carer in each case where there is one available (we will not exclude people who do not have a carer); based on previous experience [93], we anticipate that 70% will have a participating carer, giving a sample of 1050 carers.

### Recruitment

The study will run in a number of National Health Service (NHS) sites in England, Scotland and Wales, and a local Principal Investigator will oversee the conduct of the study at each site. Recruitment will be carried out by staff of the UK research networks (NIHR CRC DeNDRoN in England, NISCHR CRC in Wales, and SDCRN in Scotland), who will also conduct the questionnaire-based assessments. Participants will be recruited from Memory Services and other specialist clinics, and from databases listing people with dementia who are interested in research participation, also drawing on contacts with community mental health teams, GP practices, social services and voluntary sector groups as appropriate. Assessments will be conducted in participants’ own homes. Participating in this observational study will not preclude participation in intervention trials; any such participation in interventions will be noted. Interviews for qualitative data collection will be conducted with a subset of the sample by a dedicated interviewer, again in participants’ own homes.

### Sampling strategy

Our sampling strategy is informed by robust population estimates of the key parameters of dementia sub-type, age, gender, living situation and relationship with primary carer. To ensure that we are achieving our target sample we will examine our T1 data at pre-determined times (6 months, 12 months, 18 months) to evaluate the distribution of these parameters within our sample, along with the distribution of MMSE scores indicative of dementia severity, and adjust recruitment targets if necessary. We will assess our sample in relation to indices of social deprivation (by postcode) and, if necessary, adjust our recruitment targets to sample more intensively from areas with higher or lower social deprivation.

We have estimated an attrition rate of 30% over the course of the study but we acknowledge that this may vary according to participant characteristics and may be problematic if attrition rates are higher for sub-groups where T1 numbers are small. To address this we will monitor attrition rates in the first 6 months of T2 data collection, as collection of T2 data will begin 12 months before collection of T1 data is complete. Should we identify any unexpected differential attrition rates, we will use this information to adjust T1 recruitment targets for the remaining 6 months of T1 data collection. Attrition may occur for various reasons, some of which are amenable to amelioration. Unavoidably, there will be some deaths and cases of serious illness in people with dementia or carers that preclude further participation. Other potential causes of attrition are people moving area or moving into a care home; in this study we intend to see people who move into care homes, or who move area, wherever possible. Some people with dementia may decide they do not wish to complete further assessments; in these cases we will seek to continue to obtain information from the carers wherever possible. We will maintain contact with all participants and carers between assessments by means of a regular twice-yearly study newsletter, as well as informal telephone contact. As some people with dementia may lose capacity to consent during the course of the study we will, on entry to the study, identify a personal consultee for each participant, who can advise on continued participation if and when this becomes necessary. To minimise the effect of attrition in the planned analyses, missing data may be imputable from other obtained data. This imputation will reduce the risk of bias caused by missing data. The statistical analysis plan will include sensitivity analyses to allow us to fully understand the effect of the assumptions made in any imputation processes used [94].

It is important to ensure that the sample includes sufficient numbers for key sub-group analyses relating to age, gender, dementia sub-type, living situation (alone vs. with others), and carer relationship (spouse vs. child). While sufficient numbers are expected in most sub-groups, our estimates suggest it will be necessary to over-sample for people with Parkinson’s disease dementia and fronto-temporal dementia, and people with early-onset dementia. This will be achieved by specifically targeting movement disorders clinics where people with Parkinson’s disease dementia are seen, specialist memory clinics known to have a particular focus on fronto-temporal dementia, and specialist services for working-age individuals with dementia.

Qualitative sampling will be based on findings from the T1 stage of the quantitative study. Individuals will be identified using T1 and T2 data to construct a qualitative sample of 30 people with dementia showing positive or negative changes in indicators of living well and their 30 carers (where there are carers). Following stratification by QoL we will adopt a maximum variation sampling method aiming to include representation from people from different diagnostic groups, ethnic, social and educational backgrounds, and geographical locations. We will also aim to recruit carers to be interviewed where available.

### Measures

The questionnaire survey for quantitative analysis will relate to each component of the hypothesised model and will be completed by all participants at each time point, unless otherwise indicated. Carers will act as respondents on their own account and, where required, as informants regarding the person with dementia. The questionnaire survey has been derived from short versions of available measures, or identification of sub-scales or single items with known psychometric properties from these measures, to provide a streamlined assessment requiring two visits at each time point. The content of the survey at T1 is summarised in Table 1. In addition to personal and background details, the questionnaire survey covers *capitals, assets and resources* (social, financial, environmental, physical, psychological), access to and use of social and health care, including community resources, and quality of the caregiving relationship (where relevant); dementia-related and other *challenges*, including dementia severity, co-morbidity (with regard to both physical and mental health) and dependence; *adaptation*; and indicators of *living well* (well-being, life satisfaction, QoL, social participation, and expression of positive emotions). The survey includes a number of questionnaires, which are listed in Table 2, as well as questions drawn from existing surveys (British Social Attitudes Survey, Scottish Social Attitudes Survey, National Survey for Wales, Welsh Housing Quality Survey, UK 2011 Census, Millennium Cohort Study, CFAS, Cultural Capital and Social Exclusion Survey, English Longitudinal Study of Ageing, and Health Survey for England). For each component involving self-rating by the people with dementia, we will also either identify one or more priority questions that can continue to be administered at later time points where the person with dementia is no longer able to complete the full set of items. Where appropriate, informant ratings by the carers will be elicited alongside self-ratings to inform the interpretation of informant ratings at later time points where the person with dementia is unable to provide self-ratings, and carers will rate the expression of positive emotions in the person with dementia at each time point to facilitate interpretation of data relating to people with severe dementia. People with dementia will complete brief cognitive tests selected according to current severity of dementia. If the carer changes during the course of the study we will ask the new carer to complete the appropriate informant measures wherever possible.

**Table 1:** **Overview of main domains and topics assessed at Time 1 for people with dementia and carers**

**Domain/topic**	**Person with dementia**	**Carer**
**Personal and background details**		
Demographic information	x	x
Diagnosis	x	
**Capitals, assets and resources**		
*Social and environmental resources*		
Household income of the people with dementia	x	x
Social capital and social resources	x	x
Cultural capital	x	x
Housing and environment	x	x
Dementia-friendly community		x
*Relationships and interactions*		
Social networks and interactions	x	x
Loneliness	x	x
*Psychological traits and attitudes*		
Personality	x	x
Optimism	x	x
Self-esteem	x	x
Self-efficacy	x	x
Self-acceptance	x	
Sense of self	x	
Spirituality and religious activity	x	x
Attitudes to ageing	x	
**Challenges**		
*Psychological state*		
Mood	x	x
*Physical health and physical activity*		
Health conditions	x	x
Health state	x	x
Sensory impairment	x	x
Sleep	x	
Falls	x	x
Nutrition and appetite	x	
Alcohol use and smoking	x	x
Physical activity	x	x
*Life events*		
Life events in past 12 months	x	x
*Dementia-related issues*		
Stage and severity of dementia	x	
Cognition	x	
Functional ability	x	
Neuropsychiatric symptoms	x	
Dependence	x	
Involvement in decision-making	x	
Perceived stigma	x	
Carer stress		x
Role captivity		x
Social restriction		x
Perceived severity of neuropsychiatric symptoms in the participant		x
Distress experienced as a result of the participant’s symptoms		x
Lost employment		x
Hours of care provided		x
**Access to/use of services**		
Service receipt	x	
Satisfaction with services	x	
Extent of payment for care	x	
**Adaptation**		
Information about the condition	x	x
Understanding of the condition	x	x
Impact of the condition	x	
Coping		x
Caregiving competence		x
Positive aspects of caregiving		x
**Living well outcomes**		
Well-being	x	x
Life satisfaction	x	x
Quality of life	x	x
Activity participation	x	
Expression of positive emotions	x

**Table 2:** **Standardised questionnaires used in IDEAL at Time 1**

**Measure**	**Relating to the person with dementia**	**Relating to the carer**
**Capitals, assets and resources**		
Resource Generator - UK	x	x
Lubben Social Network Scale	x	x
Positive Affect Index	x	x
De Jong Gierveld Loneliness Scale	x	x
Mini-IPIP	x	x
Life Orientation Test - Revised	x	x
Rosenberg Self-Esteem Scale	x	x
Generalised Self-Efficacy Scale	x	x
Ryff Scales of Psychological Well-being - Self-Acceptance Subscale	x	
Philadelphia Geriatric Center Morale Scale - Attitude Toward Own Ageing Subscale	x	
Social Capital Harmonised Question Set	x	x
**Challenges**		
Geriatric Depression Scale	x	
Center for Epidemiologic Studies Depression Scale - Revised		x
Charlson Co-Morbidity Index	x	x
EQ-5D-3L	x	x
Simplified Nutrition Appetite Questionnaire	x	
General Practice Physical Activity Questionnaire	x	x
Functional Activities Questionnaire	x	
Dependence Scale	x	
Decision-making Involvement Scale	x	
Neuropsychiatric Inventory Questionnaire	x	x
Relative Stress Scale		x
Role Captivity		x
Modified Social Restriction Scale		x
**Access to and use of services**		
Client Services Receipt Inventory	x	
**Adaptation**		
Representations and Adjustment to Dementia Index	x	x
Carer Coping - Management of Situation		x
Carer Coping - Management of Meaning		x
Caregiving Competence Scale		x
Positive Aspects of Caregiving		x
**Living well outcomes**		
WHO-5 Well-being Index	x	x
Satisfaction with Life Scale	x	x
Quality of Life in Alzheimer’s Disease	x	
Activity and Affect Indicators of Quality of Life	x	
WHOQOL-BREF		x

The aim of the qualitative arm will be to gather intensive longitudinal data on the lived experiences of people with dementia and carers focusing on changes over time in challenges, assets and resources and adaptation to these changes, including illness trajectories and careers, the effects of key transitions and disruptions (e.g. moving into residential care), the depth and quality of social networks, friendships and social support and the nature of changes over time in those networks. Interviews will be undertaken at T2 and T3 of the quantitative study. Methods will incorporate qualitative social network analysis and in-depth interviewing. The research will also explore out-of-home mobility to examine the relationship between mobility, use of social space, sense of community and belonging, engagement in meaningful activities and well-being and identifying barriers to and facilitators of mobility and social participation. We will undertake in-depth qualitative interviews to examine the extent to which people with dementia and carers feel they are able to live well with dementia, their social networks, their use of social space and community resources, what helps or hinders the possibility of living well, and what factors are particularly important to them in relation to being able to live well. The interviews will include detailed exploration of individual social capital resources and network embeddedness using ego-net approaches [95]. We will employ techniques allowing for the visualisation of name generators [96,97], name interpreters and position generators, leading to measures of multi-strandedness and multiplexity. Interviews will be piloted and adapted for use with people with dementia and carers prior to the T2 assessment, and the methodological lessons learnt will be incorporated into the project outputs. The possibility of the people with dementia and their carers having divergent views will be raised when undertaking consent for interviewing. Analysis of qualitative data will address the different perspectives within the caring dyad through constant comparison and triangulation within the dyad and comparison across caring dyads for divergent cases (where there may be different forms of divergence and agreement). The research will be longitudinal in two senses: working biographically with people with dementia and carers from the point of diagnosis to the time of entry to the study, and working prospectively by undertaking interviews at T2 and T3, allowing a longitudinal perspective on the careers and pathways of people with dementia and carers. Given that not all participants will have a carer, if attrition at T3 markedly reduces the number of carers, we will augment numbers by resampling at that point.

### Procedure

People with dementia considered to meet the study inclusion criteria, with a carer where applicable, will be contacted by telephone or letter or spoken to in person during clinic appointments to establish whether they are interested in participating. Those who express interest will be sent further information and later visited at home, and where appropriate, consent will be taken from the people with dementia and from the carers if available. Non-responses to the initial contact will be followed up on one occasion by research network staff to compensate for the possibility that letters and messages could be mislaid due to memory difficulties. The T1 assessment will be conducted during two further home visits. Participants will be followed up 12 (T2) and 24 (T3) months later, completing the assessment in two home visits at each time point. An acceptable window for follow-up will be no earlier than one month prior to scheduled follow-up date and no later than two months after the scheduled follow-up date at each time point. The participants will be offered a small shopping voucher as a token of appreciation for taking part in the study upon the completion of the questionnaire-based assessment at each time point. Participants identified as meeting criteria for the qualitative interview will be contacted by a dedicated researcher, and if they are willing to engage in this element of the study this researcher will visit them at home to conduct the interview.

### Data analysis: quantitative (research questions 1 and 2)

Confirmatory analyses will test the theoretical model and examine hypothesised bivariate and multivariate relationships between factors considered to affect aspects of living well for both people with dementia and carers. Exploratory analyses using SEM will then generate extended models uniting and refining the simpler models tested earlier. Analyses will take account of symptom severity and progression and of co-morbidity. At T1 the sample will be fully described and simple relationships explored using correlational analyses. The sample characteristics will be benchmarked against existing data to assess the representativeness of key subgroups. Quantitative analyses will use multiple regression to examine relationships at T1, linear mixed models will assess changes over time and SEM will generate models to address the primary research questions. We will also seek to identify any reciprocal influences between people with dementia and carers factors and examine the interdependence of the two perspectives. The relationship between people with dementia self-ratings and carers informant ratings will be assessed to establish both the correlation and the bias (agreement) between the measures [98]. If the correlation is sufficiently high, the informant measure, corrected for the established bias, will be substituted for the self-rated measure where this is missing at T2 or T3. Sample size has been determined in relation to SEM analyses [91]. Further sensitivity analyses will explore how the models generated differ according to gender, dementia sub-type, and dementia severity. Particular attention will be paid to the robustness of the models for key subgroups where numbers permit, for example people with dementia who live alone and/or who do not have family involved in their care, and black and minority ethnic people with dementia and carers. Options for linkage to administrative data, in order to conduct further analyses, will be explored.

Health and social care costs will be calculated by attaching nationally applicable unit costs to units of reported service use. Out-of-pocket costs to people with dementia, or costs to carers related to supporting the person with dementia (e.g. for travel related to condition-specific treatment, and contribution to costs of care), will be calculated from information collected in the survey, as will be the costs of lost production and replacement costs of informal (unpaid) care. The planned analysis will examine the following questions:

1. What health and social care services are used by people with dementia?
2. What are the costs to health and social care of supporting people with dementia?
3. What are the costs to the people with dementia and their carers (e.g. out-of-pocket spend, lost income)?
4. Are health and social care services impacting on the ability of people with dementia to live well and on the ability of the carers to cope with caring responsibilities?
5. What is the relative impact of different services on the ability of people with dementia to live well, compared to the impact of dementia-related challenges and the inherent characteristics of people with dementia and carers?
6. How does the ability of people with dementia to live well change with different levels and combinations of services?
7. How does adaptation impact upon the relationship between service use and the ability of people with dementia to live well over time?

Descriptive statistics will be produced for service use and costs to health and social care and to people with dementia and carers (Q1, 2, 3). Multivariate analyses will also be conducted to explore (i) patterns of costs in relation to the demographic and needs-related characteristics of people with dementia and carers and their capitals, assets and resources and (ii) outcomes for people with dementia and for the carers in relation to patterns of service use, needs-related characteristics and other factors. Analyses will use general linear modelling of T1 data (Q4, 5) and multilevel approaches such as growth curve modelling of data over the study period (Q6, 7).

### Data analysis: qualitative (research question 3)

The aim of these analyses will be to construct illness careers and illness trajectories [99] and where possible explore explanations for variations in career trajectories using qualitative comparative analysis [100]. We will adopt a relational sociology perspective to construct case studies of people with dementia and carers addressing the background to illness onset, the interface with services and support, and social networks [101]. The research will adopt a mixed-method approach to the collection and analysis of social network data, producing network maps using appropriate software in combination with the analysis of interview narratives to unpack the meaning and feelings attached to networks as part of individual life-stories [102]. In a similar way data on the use of social space by people with dementia will be utilised and constructed in combination with analyses of individual accounts of social activities outside the home.

### Integration of quantitative and qualitative findings

We will aim to integrate the qualitative and quantitative research findings, first through the sampling process and second through the process of analysis and interpretation. In the first instance our qualitative sampling strategy will be based on identifying individuals with positive or negative changes in living well through the quantitative findings at T1 and T2. In this sense the quantitative study will form the basis for recruitment and stratification in the qualitative study. Both quantitative data and qualitative data will be longitudinal and the emphasis in the qualitative interviewing will be on exploring the careers of people with dementia and their carers and the lived experience of dementia in the context of changes over time in challenges, assets and resources and adaptation to these changes. This will allow us to explore changes in the meanings, beliefs and feelings associated with particular forms of challenges, assets and resources and where appropriate undertake further qualitative analysis to explore in depth important factors and spheres of social life identified, from the analysis of quantitative data at T2 and T3, as playing key roles in enabling people with dementia and carers to live well. This qualitative work will be used to reflect back on the quantitative analysis to explore possible explanations for patterns in the data and to inform theory building.

## Discussion

The IDEAL study is a five-year longitudinal cohort study of the experiences of 1500 people with dementia and their family carers across Great Britain which aims to determine how social, psychological circumstances and resources influence the ability to live well with dementia and identify what can be done by individuals, communities, health and social care practitioners, care providers and policy-makers to improve the likelihood of living well with dementia. By living well, we mean maximising life satisfaction, reaching one’s potential for well-being, and experiencing the best possible quality of life in the context of the challenges that dementia presents for individuals, relationships and communities. The findings will provide evidence on which to inform policy, practice and resource allocation discussions. IDEAL will generate evidence to inform developments that will have major impacts on the lives and experiences of people with dementia and carers in the UK and internationally. We aim to empower people with dementia and carers to make changes in their lives and manage the condition effectively, to provide information, knowledge and skills for practitioners that will enable them to offer early identification and diagnosis coupled with effective support, to provide an evidence-base for decisions about social policy and appropriate targeting of resources, and to educate and inform the general public about dementia in order to support the development of dementia-friendly communities, as outlined in the Prime Minister’s challenge on dementia [3]. The Alzheimer’s Society and Innovations in Dementia, as project partners, will play a key role in ensuring effective communication and implementation of findings. A project advisory group, with representation from a range of professional, clinical and patient organisations, will also meet bi-annually to review progress and shape recommendations. Results from IDEAL will be used to develop an evidence-based action plan in collaboration with key stakeholders and drawing on input from regional workshop participants, and this will be presented at an end-of-study conference. Recommendations within the action plan will highlight areas where change is possible and achievable at individual, family, community and societal levels and how such change might be achieved, and we will work collaboratively with people with dementia, carers, relevant organisations, service providers, policy makers and Government advisers to ensure uptake and implementation.

To ensure that IDEAL is relevant to the needs of people with dementia and carers, extensive consultation was undertaken when developing plans for the research. Members of the Alzheimer’s Society Research Network and of the Innovations in Dementia Think Tank of people with dementia contributed their views on the proposal and all those consulted considered that the proposed research had merit: *‘Yes, 100%*’. They also expressed readiness to participate in such a study and contributed useful perceptions of what it means to ‘live well’ with dementia: *‘To live as best I can at home with family and friends and carrying on my existing life as good as possible.*’ The issues that they identified as impacting on the ability to live well, which included loneliness, lack of understanding of dementia in their communities, losing contact with friends, isolation, fear of going out independently, and loss of control over aspects of one’s life, contributed to the decision to focus on social, environmental and psychological factors in this project. Involvement of people with dementia and carers is equally integral to the conduct and delivery of the study, and an independent project advisory network of people with dementia and carers, the Action on Living Well: Asking You (ALWAYS) group, is available for consultation and is represented on the Project Advisory Group. Members of the ALWAYS group have contributed to questionnaire design and staff training materials, and will continue to comment on the conduct of the project and contribute to interpretation of findings, preparation of the action plan, and the best ways of presenting the findings to people with dementia, carers and the general public.

Information about IDEAL is available on the project website [103] which will act as a focal point for findings to be collated and disseminated to the public and practitioners. IDEAL, the first large-scale study of its kind, offers a unique resource for social science research in the UK and internationally.
